# The battle against pediatric retinoblastoma: insights from 2024 medical literature

**DOI:** 10.1016/j.htct.2025.103839

**Published:** 2025-08-08

**Authors:** Yoshiyasu Takefuji

**Affiliations:** Faculty of Data Science, Musashino University, Tokyo, Japan

This literature review, based on 2024 peer-reviewed publications from the National Library of Medicine, provides a concise overview of retinoblastoma, a pediatric cancer that necessitates multidisciplinary care. The primary objectives of treatment are to preserve life and vision [1]. Extraocular retinoblastoma can likely be cured with systemic chemotherapy and radiation therapy. In contrast, extra-orbital disease demands intensive chemotherapy, potentially consolidated with high-dose chemotherapy and autologous hematopoietic stem cell rescue, with or without radiation therapy [1]. Retinoblastoma, although relatively uncommon and accounting for about 3% of cancers in children under 15 years, is a significant concern [1]. It originates from the retina and may grow under the retina or towards the vitreous cavity. Most patients present with leukocoria, often first noticed after a flash photograph is taken. Screening guidelines have been established for children at risk of developing retinoblastoma [1].

As the most common pediatric ocular malignancy, retinoblastoma is typically caused by a mutation in the *RB1* gene or *MYCN* oncogene amplification [2]. The disease can be unilateral (60%–70%) or bilateral (30%–40%), with hereditary cases being bilateral and appearing earlier than unilateral ones, especially in India. Outcomes in developing countries are worse due to high prevalence, delayed presentation, and healthcare inaccessibility. Treatment has evolved from enucleation and radiotherapy to less aggressive eye salvage modalities. Multimodality treatment, including chemotherapy and focal consolidation therapies, is now standard. Genetic testing, counseling, and screening are essential, with advances in genetics paving the way for potential targeted therapy [2].

Addressing *RB1*-deficient cancers presents a challenge. Dysfunctional or deleted *RB1* can enhance cancer proliferation and spread [3]. Scientists are striving to understand the role of *RB1* in various biological processes and molecular pathways to develop innovative therapies for *RB1*-deficient cancers. Recent advancements have shown promising strategies for combating these cancers, leading to more precise and effective treatments. They emphasized the crucial role of *RB1* in cancer research and its potential for personalized therapies. *RB1*, a critical tumor suppressor gene, plays a significant role in cell cycle control and DNA repair mechanisms, restraining abnormal cell growth and maintaining genomic stability. Understanding the complex interactions of *RB1* with cellular pathways is key to developing targeted therapies [3].

Retinoblastoma, a prevalent pediatric vision-threatening condition, has traditionally been treated with conventional therapies like systemic chemotherapy and focal therapy [4]. However, the quest for tumor eradication with minimal impact on surrounding tissues continues. Researchers explored the genetic origin, classification, and treatment modalities with their combination with nano-scale delivery systems for tumor targeting. They also discussed ongoing clinical trials, patents, and emerging therapies like gene therapy and immunotherapy. Understanding genetics in retinoblastoma development has refined treatment strategies. The novel approaches discussed here could overcome limitations of conventional therapy, thereby improving retinoblastoma treatment outcomes [4].

Balaji et al. reported that retinoblastoma is a pediatric eye cancer occurring in 1/15000 live births [5]. Initiated by *RB1* gene inactivation, its progression relies on transcriptional changes. This study used RT2 Profiler™ PCR array to analyze 84 cancer-specific genes, identifying 68 dysregulated genes. Key alterations were validated by real time quantitative reverse transcription polymerase chain reaction (RT-qPCR) and Western blot, revealing potential therapeutic targets. Polymerase chain reaction (PCR) arrays are suggested for rapid, cost-effective gene expression analysis in retinoblastoma [5].

Cobrinik et al. reported that retinoblastoma is a childhood retinal cancer affecting six out of 100,000 live births, with 250–300 new cases annually in the U.S. and 8000 worldwide [6]. Early detection often leads to a cure, with advanced cases posing clinical challenges. Initiated by *RB1* gene inactivation, recent studies have identified the cell of origin and genomic changes linked to treatment resistance. This understanding may improve therapies and prevention for genetically predisposed children [6].

Zhou et al. states that retinoblastoma, a primary ocular malignancy in children that without prompt treatment poses a significant mortality risk [7]. Survival and vision preservation depend on disease severity at diagnosis. Initiated by *RB1* gene mutations, tumorigenesis involves genetic and epigenetic changes. The *RB1* gene encodes a protein crucial for cell replication control. Diagnosis and treatment consider disease stage, germline mutation status, psychosocial factors, and institutional resources, enhancing care quality for this pediatric cancer [7].

Singh et al. concluded that retinoblastoma is the most common childhood intraocular tumor, caused by *RB1* gene mutations on chromosome 13 [8]. Annually, 8000 children are diagnosed globally, with 1500 cases in India. Survival rates exceed 90% in developed countries. Leukocoria and proptosis are common in Asian Indian populations. Diagnosis involves fundus examination and ultrasound. Prenatal and genetic testing benefit high-risk families. Histopathologic risk factors predict metastasis, requiring aggressive treatment for advanced cases [8].

Rabelo et al. reported that retinoblastoma, the most common childhood intraocular tumor, faces diagnostic and treatment challenges, especially in low- and middle-income countries [9]. Improving time to diagnosis and treatment access is crucial. A systematic review identified 21 studies with potential interventions, categorized into surveillance, genetic counseling, education, public assistance, and international partnership. These initiatives aim to enhance diagnosis and treatment access, proposing a comprehensive chain of initiatives to improve clinical outcomes for retinoblastoma patients [9].

The literature review provides a comprehensive overview of retinoblastoma, a significant pediatric cancer requiring multidisciplinary care. It emphasizes the primary treatment objectives of preserving life and vision, discussing various modalities such as systemic chemotherapy, radiation therapy, high-dose chemotherapy, and autologous hematopoietic stem cell rescue. The review delves into the genetic causes, particularly *RB1* gene mutations and *MYCN* oncogene amplification, and highlights the challenges faced in developing countries due to high prevalence, delayed presentation, and healthcare inaccessibility. It notes the evolution of treatment from enucleation and radiotherapy to less aggressive eye salvage modalities and multimodality treatment, stressing the importance of genetic testing, counseling, and screening. The review also addresses the challenges of *RB1*-deficient cancers and ongoing research to develop innovative therapies, including gene therapy, immunotherapy, and nano-scale delivery systems. Additionally, it mentions ongoing clinical trials and patents, diagnostic and treatment challenges, especially in low- and middle-income countries, and suggests potential interventions to improve clinical outcomes. Overall, the review provides an up-to-date understanding of retinoblastoma, its genetic basis, treatment options, and research efforts to enhance patient outcomes.

## Author contribution

Yoshiyasu Takefuji completed this research and wrote this article.

## Conflicts of interest

The author declares no conflicts of interest.
